# Contributions to the knowledge of the genus *Horaeomorphus* Schaufuss (Coleoptera, Staphylinidae, Scydmaeninae) in mainland China

**DOI:** 10.3897/zookeys.572.7474

**Published:** 2016-03-15

**Authors:** De-Yao Zhou, Su-Jiong Zhang, Li-Zhen Li

**Affiliations:** 1 Department of Biology, College of Life and Environmental Sciences, Shanghai Normal University, 100 Guilin Road, Shanghai, 200234, P. R. China; 2 Forestry Bureau of Pan’an County, Pan’an 322300, Zhejiang, China

**Keywords:** Scydmaeninae, Glandulariini, *Horaeomorphus*, new species, new records, Oriental, China

## Abstract

Five new species of the ant-like stone beetle genus *Horaeomorphus* Schaufuss (Scydmaeninae: Glandulariini) from China are described: *Horaeomorphus hainanicus* **sp. n.**, *Horaeomorphus biwenxuani* **sp. n.**, *Horaeomorphus pengzhongi* **sp. n.**, *Horaeomorphus hujiayaoi* **sp. n.** and *Horaeomorphus punctatus* **sp. n.** The previously unknown male of *Horaeomorphus chinensis* Franz, 1985 is now discovered, and its aedeagus and metatrochanter are illustrated. The latter species is newly recorded from Zhejiang. Three females from Guangxi are also recorded, but their identity remains unconfirmed until associated males become available. A key to *Horaeomorphus* of mainland China is included.

## Introduction

The Australo-Oriental genus *Horaeomorphus* Schaufuss currently comprises 59 species distributed in Malaysia, Singapore, Thailand, Nepal, Vietnam, Japan, continental China, Taiwan, Laos, the Philippines, Indonesia, Fiji, Australia, and Madagascar (Franz 1985; Jałoszyński 2002, 2003, 2004, 2006, 2009, 2012, 2014a, 2014b; Jałoszyński and Nomura 2004, 2008; Jałoszyński et al. 2007; Vít 2004); among them 21 Madagascan species were transferred from previous Euconnus (Anthicimorphus) together with the Australian type species of *Anthicimorphus*, but they should be treated as species *incertae sedis* within Glandulariini (Jałoszyński 2014b). Until now, only one species from mainland China has been described.

Among asian glandulariine (= former Cyrtoscydmini, see Newton 2015) genera, *Horaeomorphus* is characterized by the mesoventral intercoxal process being shorter and less elevated than the mesocoxae, the metaventral intercoxal process with two long spines projecting posteriorly, the presence of a small pit at the posteromesal margin of each supra-antennal tubercle, pronotal base with distinct median pit in addition to lateral pits, and each elytron with two foveae connected by a U-shaped groove extending anteriorly; many species have the male trochanters modified (Jałoszyński 2015).

Recent examination of unsorted material in our collection revealed five new species of *Horaeomorphus* from Hainan, Guangxi, Yunnan and Xizang. Moreover, during our recent expedition, two males of *Horaeomorphus chinensis* Franz, 1985 were discovered in the type locality, Fujian: Guadun (=Kuatun), which makes it possible to add a description of male characters. This species is also newly recorded from Zhejiang: Baishanzu. Three females from Guangxi are recorded; they belong to a group of species characterized by a strongly convex and broad body, but their identities remain unknown until associated males become available. A key to *Horaeomorphus* of mainland China is included.

## Material and methods

All material treated in this study is housed in the Insect Collection of Shanghai Normal University, Shanghai, China (SNUC).

The collecting data are quoted verbatim. Each type specimen bears the following label: ‘HOLOTYPE [red] (or PARATYPE [yellow]), ♂ (or ♀), *Horaeomorphus* + specific name sp. n., det. Zhou & Zhang, 2016, SNUC’.

The following abbreviations are applied: AeL—length of the median lobe of aedeagus in ventral view; AnL—length of the antennae; BL—length of the body (= HL + PL + EL); EI—elytral index (= EL / EW); EL—length of the elytra along the suture, from the base of scutellum to the apex; EW—maximum width of the elytra; HW—width of the head across eyes; HL—length of the head from the anterior clypeal margin to the occipital constriction; PL—length of the pronotum along the midline; PWb—width of the pronotum at base; PWm—maximum width of the pronotum; SpL—length of the spermatheca.

## Taxonomy

### Horaeomorphus chinensis

Taxon classificationAnimaliaColeopteraStaphylinidae

Franz, 1985

**Figure 1.**
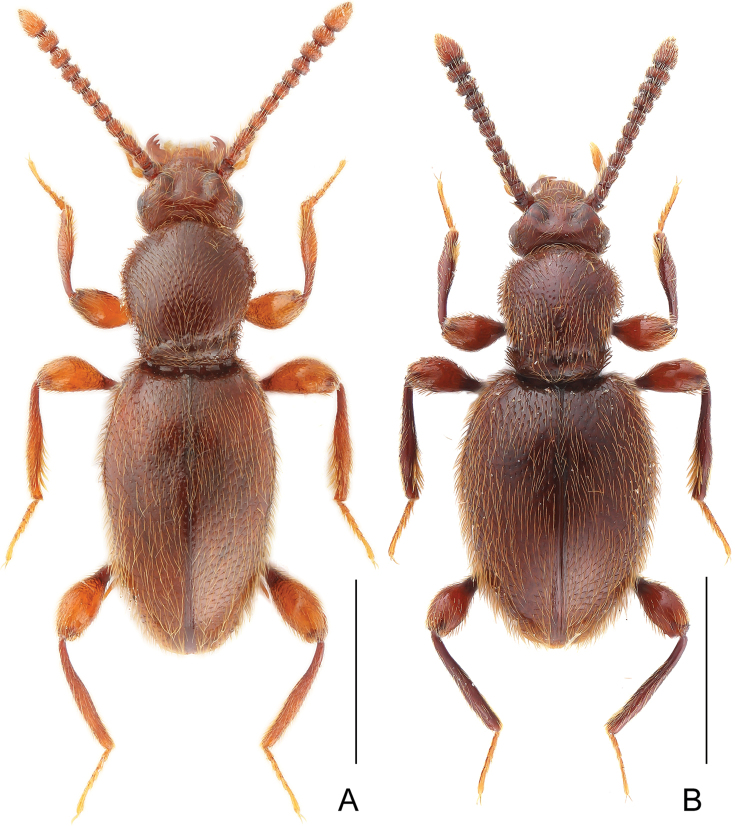
Habitus of *Horaeomorphus* species. **A** *Horaeomorphus chinensis*, male **B** *Horaeomorphus* sp., female. Scale bars: 1.0 mm.

Figs 1A , 6B–E , 6G–K

Horaeomorphus chinensis Franz, 1985: 116.

#### Material examined.

2 ♂♂, 4 ♀♀, labeled ‘China: Fujian Province, Wuyishan City [武夷山市], Tongmu Village [桐木村], Guadun [挂墩], 27°44'03"N, 117°38'40"E, decaying log, 1178m, 2.x.2015, Yan,Tu, Shen, Jiang& Zhou leg.’; 2 ♂♂,2 ♀♀, labeled ‘China: Zhejiang, Lishui City [丽水市], Qingyuan Hsien [庆元县], Baishanzu [百山祖], Huangpi Swamp [黄皮湿地], 27°49'38"N, 119°11'29"E, decaying log, 1429m, 25.iv.2015, Song&Yan leg.’; 1 ♀, labeled ‘China: S.Zhejiang, Qingyuan, Mt nr. Liyang Village [栗洋村], 27°49'38"N, 119°11'22"E, leaf litter, sifted, 29.iv.2014, 990–1160m, Peng, Song, Yan&Yu leg.’.

#### Description of male.

Males (Fig. 1A) similar to females in external morphology, but metatrochanter (Fig. 6J) modified, with distal part protruded; slightly longer than half of metafemur, apex spiculate. BL 2.52–2.76 mm; HL 0.40–0.43 mm; HW 0.53–0.58 mm; AnL 1.05–1.17 mm, relative lengths of antennomeres: 1.0 : 0.9 : 1.4 : 1.1 : 1.1 : 1.0 : 1.0 : 1.0 : 1.1 : 1.1 : 2.0; PL 0.75–0.81 mm; PWb 0.46–0.52 mm; PWm 0.65–0.71 mm; EL 1.37–1.53 mm; EW 0.89–0.94 mm; EI 1.54–1.63.

Aedeagus (Fig. 6C–E) elongate, AeL 0.48 mm; endophallus (Fig. 6H–I) very complicated, with large bell-shaped central complex surrounded at each side by weakly sclerotized, elongate lateral structures; parameres (Fig. 6G) slender, longer than median lobe, each with three apical setae and two subapical setae.

#### Distribution.

Eastern China: Fujian, Zhejiang (new provincial record).

#### Comments.

*Horaeomorphus chinensis* shares many characters with several congeners: *Horaeomorphus punctifrons* Jałoszyński, 2006 (Malaysia: Pahang), *Horaeomorphus jeraianus* Jałoszyński, 2006 (Malaysia: Kedah), *Horaeomorphus jaechi* Jałoszyński, 2006 (Malaysia: Sarawak), *Horaeomorphus tiomanensis* Jałoszyński, 2006 (Malaysia: Tioman Is.), *Horaeomorphus caverniventris* Jałoszyński, 2006 (Malaysia: Pahang), *Horaeomorphus endauensis* Jałoszyński & Nomura, 2007 (Malaysia: Pahang), *Horaeomorphus imitator* Jałoszyński, 2009 (the Philippines: Mindanao), *Horaeomorphus solodovnikovi* Jałoszyński, 2014 (Laos: Champasak), *Horaeomorphus sakishimanus* Jałoszyński, 2002 (Japan: Iriomote-jima and Ishigaki-jima islands), *Horaeomorphus hainanicus* sp. n. (China: Hainan) and *Horaeomorphus pengzhongi* sp. n. (China: Yunnan), all of them have elongate and convex habitus, the pronotum with three pits connected by a groove, the base of elytra barely wider than the basal margin of pronotum and protruded, recurved metatrochanters in males. Among these species, *Horaeomorphus hainanicus* sp. n. and *Horaeomorphus pengzhongi* sp. n. have a similar shape of the aedeagus, structures of endophallus and slender parameres each with two subapical setae, as those in *Horaeomorphus chinensis*. The character combination of the pronotum and elytra with a dense and distinct punctation, the recurved metatrochanters without expansions on their ventral margins in male, the parameres longer than median lobe and the structures of the endophallus can be used to distinguish *Horaeomorphus chinensis* from the two species mentioned above.

#### Bionomics.

Specimens from Guadun were collected by sifting material from an ant nest (Fig. 10B) in a relatively dry rotten trunk in a bamboo forest; four specimens from Baishanzu were collected from under bark of a rotten tree.

### Horaeomorphus sp.

Taxon classificationAnimaliaColeopteraStaphylinidae

Figs 1B , 6A , 6F

#### Material examined.

3 ♀♀, labeled ‘China: Guangxi Prov., Shangsi County [上思县], Shiwandashan [十万大山], alt. 300–400m, 23.iv.2011, Peng, Zhai &Zhu leg.’.

#### Description.

Body (Fig. 1B) large, strongly convex, BL 2.48–2.51 mm; HL 0.37–0.38 mm; HW 0.50 mm; antennae (Fig. 6A) relatively short, AnL 0.99 mm; relative lengths of antennomeres: 1.0 : 0.8 : 1.2 : 1.0 : 1.0 : 0.9 : 0.9 : 0.9 : 1.0 : 1.0 : 2.2; PL 0.74–0.76 mm; PWm 0.59–0.61 mm; PWb 0.46–0.47 mm; EL 1.35–1.39 mm; EW 1.00–1.03 mm; EI 1.30–1.38.

Spermatheca (Fig. 6F) elongate, SpL 0.13mm; with longitudinal groove at middle.

#### Comments.

These females have strongly convex and broadened elytra (EI 1.30–1.38), distance between humeral calli wider than the width of the pronotum at base, large and deep punctures sharply delimited from background in the center of pronotal disc and anterior third of elytra. The shape of the spermatheca is similar to that of *Horaeomorphus caverniventris* Jałoszyński, 2006 (Malaysia: Pahang), but females of the Malaysian species are larger (2.79–3.12 mm). Therefore the three specimens almost certainly belong to a new species, but a male must be found for formal description.

#### Distribution.

Southern China: Guangxi.

### Horaeomorphus hainanicus

Taxon classificationAnimaliaColeopteraStaphylinidae

D.-Y. Zhou & S.-J. Zhang sp. n.

http://zoobank.org/754810B2-86FD-4684-8B7C-A4981F223105

Figs 2 , 4

#### Type material

(20 ♂♂, 25 ♀♀)**. Holotype: CHINA**: ♂, labeled ‘China: Hainan, Ledong Hsien [乐东县], Jianfengling [尖峰岭] N.R., Mingfenggu [鸣凤谷], 18°44'30"N, 108°50'29"E, rainforest, decaying log from a colony termite nest, 995 m, 23.i.2015, Peng, Yin, Tu, Song, Shen, Zhou, Yan, Wang leg.’. **Paratypes**: 7 ♂♂, 14 ♀♀, same locality as holotype; 1 ♀, same locality as holotype, except ‘alt. 950m, 30-IV-2012, PAN Y.H. & LI W. R. leg.’; 6 ♂♂, 7 ♀♀, labeled ‘China: Hainan, Qiongzhong Hsien [琼州县], Limu Mt [黎母山], nr. residence, path to peak, 19°10'04"N, 109°44'45"E, decaying log, 1000 m, 31.i.2015, Peng, Yin,Tu, Song, Shen, Zhou, Yan, Wang leg.’; 2 ♂♂, 1 ♀, same locality as previously except ‘01.ii.2015’; 1 ♂, 1 ♀, same locality as previously except ‘nr. residence, 19°10'04"N, 109°44'45"E, decaying log, 625m, 29.i.2015’; 1 ♂, same locality as previously except ‘path to Limu Temple, 19°08'09"N, 109°45'46"E, 580–760m, 29.i.2015, Peng, Yin, Tu, Song, Shen leg.’; 2 ♂♂, 1♀, labeled ‘China: Hainan, Wuzhi Shan [五指山] N. R., nr.reservoir, 18°53'10"N, 109°36'11"E, 500m, 22.iv.2012, leaf litter, sifted, Peng& Dai leg.’.

#### Diagnosis.

*Horaeomorphus hainanicus* can be readily separated from all other congeners by its moderately large (2.53–3.08 mm) and elongate body, fine punctation on pronotal disc and elytra, rounded apices of metatrochanters in male, parameres each with 3–5 apical setae and two subapical setae, unique structure of endophallus and shape of spermatheca.

#### Description.

**Figure 2.**
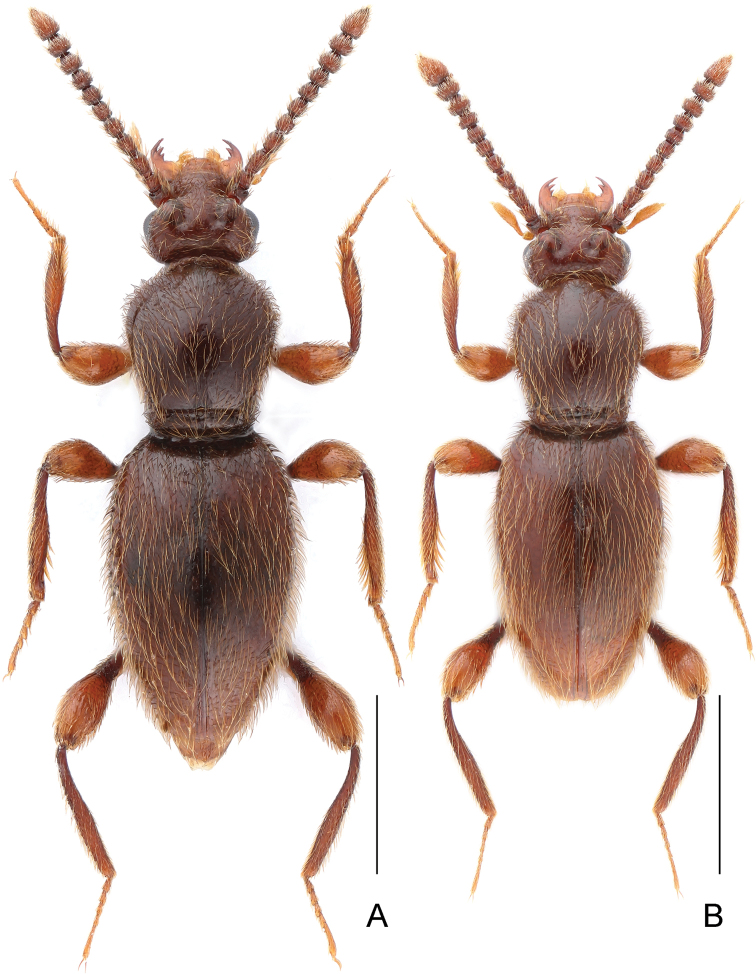
Habitus of *Horaeomorphus hainanicus* sp. n. **A** male **B** female. Scale bars: 1.0 mm.

Male. BL 2.77–3.08 mm; body (Fig. 2A) large, strongly convex, reddish brown, legs and palpi slightly lighter. Head broadest at large, finely faceted and moderately convex eyes, HL 0.40–0.48 mm, HW 0.58–0.68 mm; tempora rounded and slightly shorter than eye in dorsal view; vertex strongly transverse and weakly convex, with pair of small but distinct pits located near posterior margins of supra-antennal tubercles; frons weakly convex; supra-antennal tubercles strongly raised. Punctation on vertex and frons fine and inconspicuous; setae moderately long, sparse. Antennae (Fig. 4A) short, AnL 1.21–1.24 mm, relative lengths of antennomeres: 1.0 : 1.1 : 1.7 : 1.5 : 1.4 : 1.5 : 1.2 : 1.1 : 1.2 : 1.1 : 2.2. Pronotum inversely subtrapezoidal, widest near anterior third, PL 0.87–0.94 mm, PWm 0.72–0.81 mm, PWb 0.52–0.58 mm; anterior margin rounded, sides narrowing toward base; hind angles obtuse and blunt; posterior collar delimited from disc by deep and narrow transverse groove connecting three small pits. Punctation on disc as fine as that on frons and vertex; dorsal surface glossy; setation moderately long. Elytra oval and elongate, moderately convex; widest near anterior third, narrowing toward apices. EL 1.50–1.66 mm, EW 0.92–1.04 mm, EI 1.59–1.63. Humeral calli distinct. Punctures more distinct than those on pronotum, sharply marked and separated by spaces 3–4× as wide as puncture diameters; setation moderately dense. Hindwings fully developed. Metatrochanter (Fig. 4I) modified, with distal part protruded and recurved; as long as half of metafemur, apex rounded. Aedeagus (Fig. 4B–D) elongate, AeL 0.53 mm; endophallus (Fig. 4F–G) very complicated, with large bell-shaped central complex surrounded at each side by weakly sclerotized, elongate lateral structures; parameres (Fig. 4E) slender, longer than median lobe, each with 3–5 apical setae and two subapical setae.

Female. Similar to male, with slightly smaller body and unmodified metatrochanter. BL 2.53–2.85 mm, HL 0.39–0.42 mm, HW 0.57–0.62 mm, AnL 1.11–1.20 mm, relative lengths of antennomeres: 1.0 : 1.1 : 1.5 : 1.4 : 1.4 : 1.3 : 1.1 : 1.1 : 1.2 : 1.2 : 2.2. PL 0.76–0.88 mm, PWm 0.63–0.74 mm, PWb 0.49–0.54 mm; EL 1.39–1.54 mm, EW 0.84–0.97 mm, EI 1.58–1.64, spermatheca (Fig. 4H) ovoid, slightly elongate, SpL 0.12 mm.

#### Comments.

*Horaeomorphus hainanicus* is similar to many congeners (see comments at *Horaeomorphus chinensis*), and has a similar endophallic structures and the shape of spermatheca as *Horaeomorphus chinensis*. The relatively larger body (2.77–3.08 mm in males), the pronotal and elytral surface glossy, protruded metatrochanters with rounded apices and subtle differences in the endophallus can be used to readily separate this species from *Horaeomorphus chinensis*.

#### Bionomics.

Specimens of the type series were collected from ant and termite nest material in rotten wood and under bark of standing rotten logs in rainforests of Hainan.

#### Distribution.

Southern China: Hainan.

#### Etymology.

The specific epithet refers to the province where the type locality of the new species lies.

### Horaeomorphus punctatus

Taxon classificationAnimaliaColeopteraStaphylinidae

D.-Y. Zhou & S.-J. Zhang sp. n.

http://zoobank.org/B832FF57-E3B1-44C3-9D4F-87C3471AA98B

Figs 3 , 5

#### Type material

(1 ♂, 1 ♀)**. Holotype: CHINA**: ♂, labeled ‘China: Yunnan Prov., Yingjiang Hsien [盈江县], Xima [昔马], 1500–1650m, 20–22.v.2013, Wen-Xuan Bi leg.’. **Paratype**: 1 ♀, same locality as holotype.

#### Diagnosis.

*Horaeomorphus punctatus* can be readily separated from all other congeners by its moderately large (2.07–2.36 mm) and stout body, broad pronotum with a row of three pits connected by a shallow, barely notable groove; dense and coarse punctation on elytra, unmodified metatrochanters in male, aedeagal parameres lacking setae at apices and shape of spermatheca.

#### Description.

**Figure 3.**
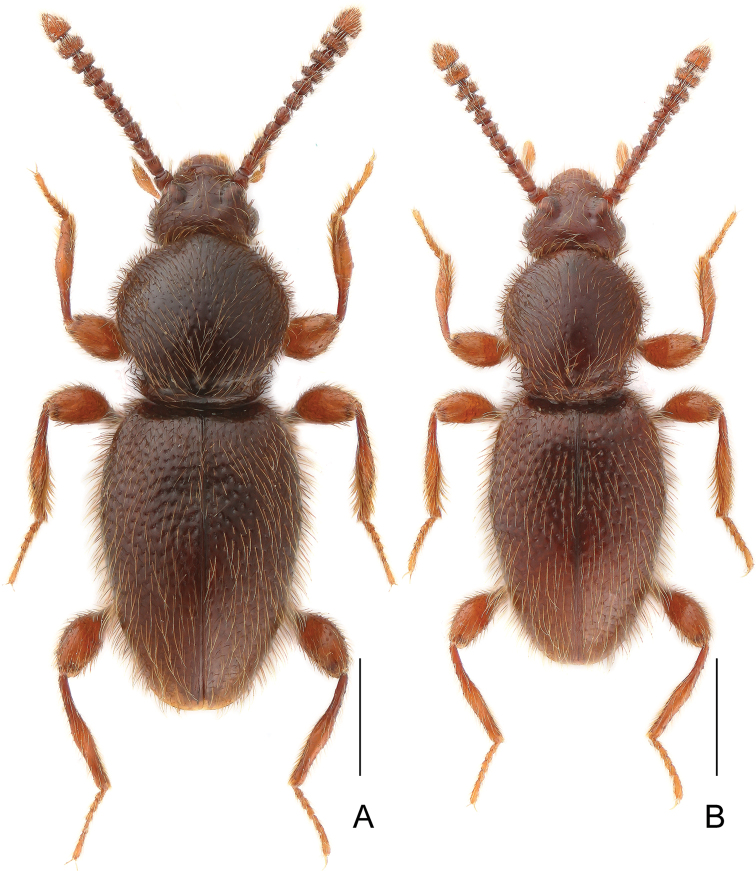
Habitus of *Horaeomorphus punctatus* sp. n. **A** male **B** female. Scale bars: 0.5 mm.

Male. BL 2.36 mm; body (Fig. 3A) moderately large, flattened, dark reddish-brown, legs and palpi slightly lighter. Head broadest at large, finely faceted and moderately convex eyes, HL 0.34 mm, HW 0.46 mm; tempora shorter than eye in dorsal view, in anterior third nearly parallel, then strongly bent and in posterior third nearly transverse to long axis of head; vertex strongly transverse and weakly convex, with pair of small but distinct pits located near posterior margins of supra-antennal tubercles; frons weakly convex; supra-antennal tubercles strongly raised. Punctures on vertex and frons fine and inconspicuous; setation moderately long, sparse. Antennae (Fig. 5A) short, AnL 0.87 mm, relative lengths of antennomeres: 1.0 : 0.9 : 1.0 : 0.9 : 0.9 : 0.8 : 0.8 : 0.8 : 0.9 : 1.0 : 1.5. Pronotum broad, nearly circular, slightly longer than wide, widest at middle, PL 0.76 mm, PWm 0.72 mm, PWb 0.47 mm; posterior collar short, well delimited from disc by constriction and row of three shallow pits connected by shallow groove; punctation dense and coarse; setation moderately long. Elytra broad, flattened and distinctly impressed in middle at about anterior third; EL 1.26 mm, EW 0.84 mm, EI 1.50. Punctures coarse, more distinct than those on pronotum, sharply marked and separated by spaces as wide as diameters of punctures; setation long, moderately dense, erect to suberect. Hindwings fully developed. Metatrochanter (Fig. 5I) unmodified, with distinct ventral edge. Aedeagus (Fig. 5B–D) relatively slender, with median lobe strongly narrowing toward apex, AeL 0.57 mm; endophallus (Fig. 5F–G) relatively small and complicated, with sclerotized central portion and two moderately darkly sclerotized and curved structures; parameres (Fig. 5E) slender, minimally longer than median lobe, without apical setae.

**Figure 4.**
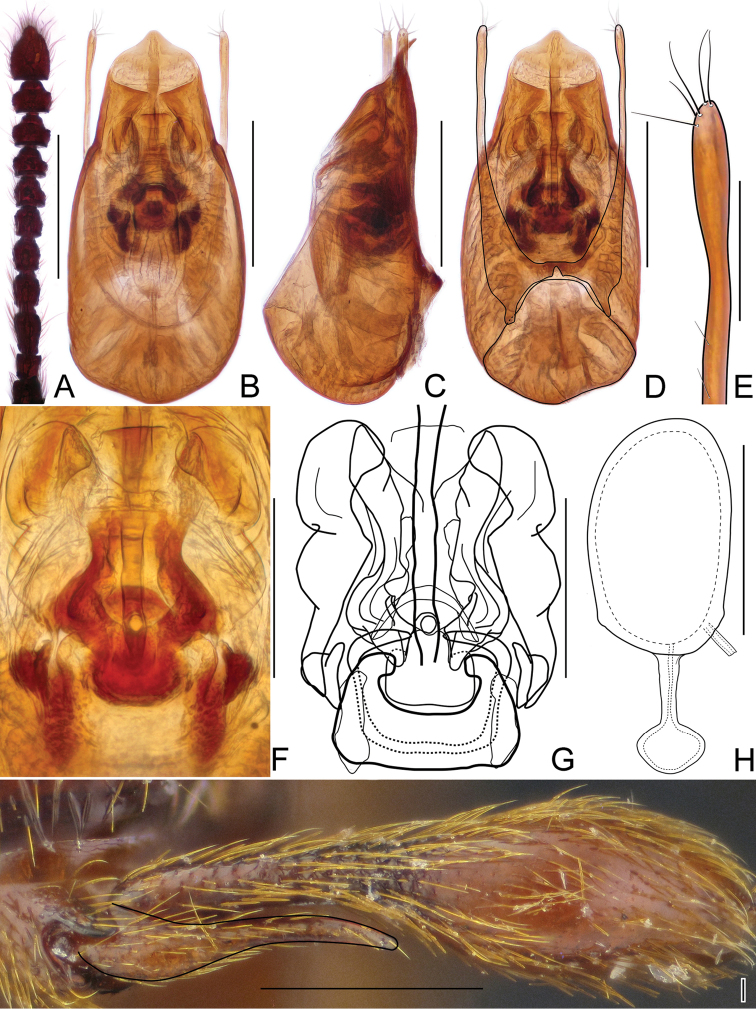
Diagnostic characters of *Horaeomorphus hainanicus* sp. n. **A** Left antenna of male, in dorsal view **B** Aedeagus, in ventral view **C** Same, in lateral view **D** Same, in dorsal view **E** Apical portion of paramere, enlarged **F** Endophallus, enlarged, in ventral view **G** Same, schematic **H** Spermatheca, in lateral view **I** Left metatrochanter of male, in ventral view. Scale bards: 0.5 mm (**A**); 0.2 mm (**B, C, D**); 0.04 mm (**E**); 0.1 mm (**F, G, H**); 0.3 mm (**I**).

**Figure 5.**
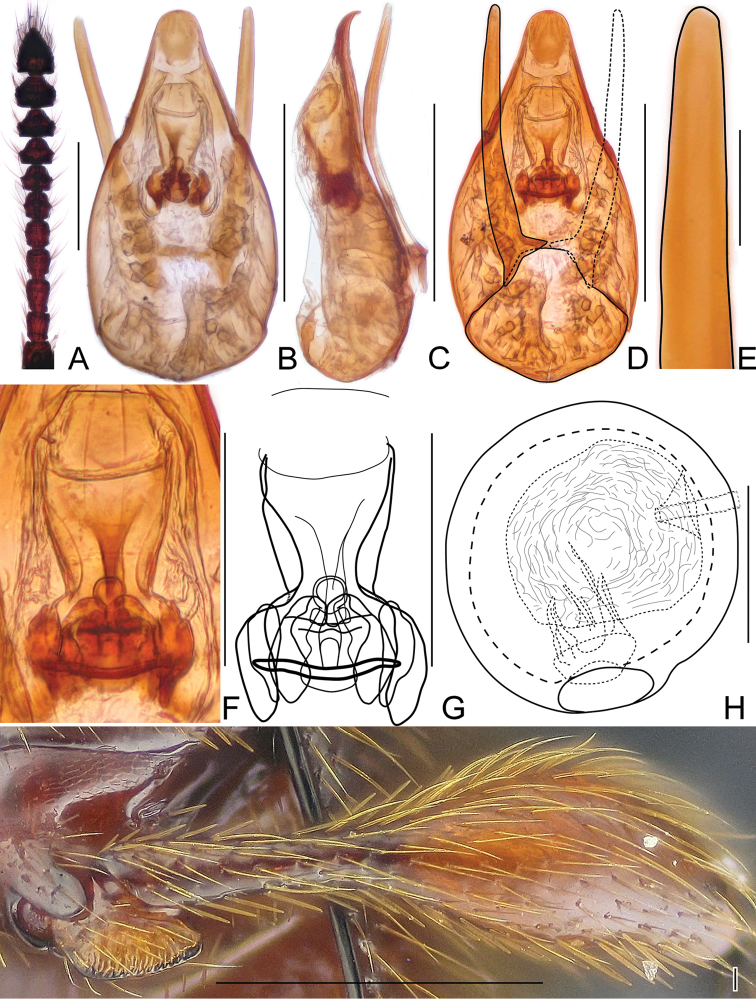
Diagnostic characters of *Horaeomorphus punctatus* sp. n. **A** Left antenna of male, in dorsal view **B** Aedeagus, in ventral view **C** Same, in lateral view **D** Same, in dorsal view **E** Apical portion of paramere, enlarged **F** Endophallus, enlarged, in ventral view **G** Same, schematic **H** Spermatheca, in lateral view **I** Left metatrochanter of male, in ventral view. Scale bars: 0.3 mm (**A**); 0.2 mm (**B, C, D**); 0.03 mm (**E**); 0.08 mm (**F, G**); 0.04 mm (**H**); 0.3 mm (**I**).

Female. Similar to male, but with smaller body and less contractive pronotal base. BL 2.07 mm, HL 0.33 mm, HW 0.44 mm, AnL 0.75 mm, relative lengths of antennomeres: 0.8 : 0.8 : 0.9 : 0.8 : 0.7 : 0.7 : 0.6 : 0.7 : 0.6 : 1.0 : 1.2. PL 0.63 mm, PWm 0.58 mm, PWb 0.44 mm; EL 1.11 mm, EW 0.74 mm, EI 1.50, spermatheca (Fig. 5H) spherical, SpL 0.08 mm, with complicated internal structures.

#### Comments.

*Horaeomorphus punctatus* is similar to *Horaeomorphus mesaios* Jałoszyński & Nomura, 2004 (Vietnam: Ninh Pinh), *Horaeomorphus valdepunctatus* Franz, 1984 (Malaysia: Pahang), *Horaeomorphus sarawakensis* Franz, 1992 (W Malaysia) and *Horaeomorphus samosirensis* Jałoszyński, 2009 (Indonesia: Sumatra) in habitus, and is also very similar to *Horaeomorphus mesaios* in the shape of aedeagus and structures of endophallus; but it can be identified by its relatively large body (2.07–2.36 mm), the pronotum with three basal pits; unmodified metafemora, non-protruded metatrochanters in males and achaetous parameral apex.

#### Distribution.

Southern China: Yunnan.

#### Etymology.

The specific epithet refers to the coarse punctation of the elytra.

### Horaeomorphus pengzhongi

Taxon classificationAnimaliaColeopteraStaphylinidae

D.-Y. Zhou & S.-J. Zhang sp. n.

http://zoobank.org/F7F96BFA-C563-45EF-AFCF-5F6539869D30

Fig. 7

#### Type material

(1 ♂)**. Holotype: CHINA**: ♂, labeled ‘China: Yunnan, Baoshan City [保山市], Tengchong County [腾冲县], Mingguang Town [明光镇], Zizhi Village [自治乡], 25°18'24"N, 98°48'22"E, 1230 m, 24.vi.2013, Dai, Song& Peng leg.’.

#### Diagnosis.

*Horaeomorphus pengzhongi* can be readily separated from all other congeners by its moderately large (3.00 mm) and elongate body, sparse and fine punctation on pronotal disc and elytra, protruded metatrochanter with expansion on its ventral edge in male, parameres each with four apical setae and two subapical setae and unique structures of endophallus.

#### Description.

Male. BL 3.00 mm; body (Fig. 7A) large, strongly convex, reddish brown, legs and palpi slightly lighter. Head broadest at finely faceted and slightly convex eyes, HL 0.47 mm, HW 0.63 mm; tempora rounded, about as long as length of eye in dorsal view; vertex strongly transverse and weakly convex, with pair of small but distinct pits located near posterior margins of supra-antennal tubercles; frons weakly convex; supra-antennal tubercles strongly raised. Punctures on vertex and frons dense and coarse; setae moderately long, sparse. Antennae (Fig. 7B) short, AnL 1.31 mm, relative lengths of antennomeres: 1.0 : 0.8 : 1.8 : 1.4 : 1.1 : 1.1 : 1.0 : 1.1 : 1.2 : 1.1 : 2.2. Pronotum oval, convex, distinctly longer than wide, widest near anterior 2/5, PL 0.97 mm, PWm 0.79 mm, PWb 0.53 mm; anterior margin rounded, sides narrowing toward base; hind angles obtuse and blunt; base with 3 large and deep pits connected by narrow groove. Punctation on disc sparse and fine; dorsal surface glossy; setation moderately long. Elytra oval and elongate, moderately convex; widest near anterior 2/5, narrowing toward apices. EL 1.57 mm, EW 1.0 mm, EI 1.56. Humeral calli distinct. Punctures fine, more distinct than those on pronotum, separated by spaces 3–4× as wide as puncture diameters; setation moderately dense. Hindwings fully developed. Metatrochanter (Fig. 7I) modified, with distal portion straight and apical 1/7 recurved, as long as half of metafemur, with expansion on ventral margin, apex rounded. Aedeagus (Fig. 7C–E) elongate, AeL 0.53 mm; endophallus (Fig. 7G–H) very complicated, with large bell-shaped central complex surrounded at each side by weakly sclerotized, elongate lateral structures; parameres (Fig. 7F) slender, subequal in length to median lobe, each with four apical setae and two subapical setae.

**Figure 6.**
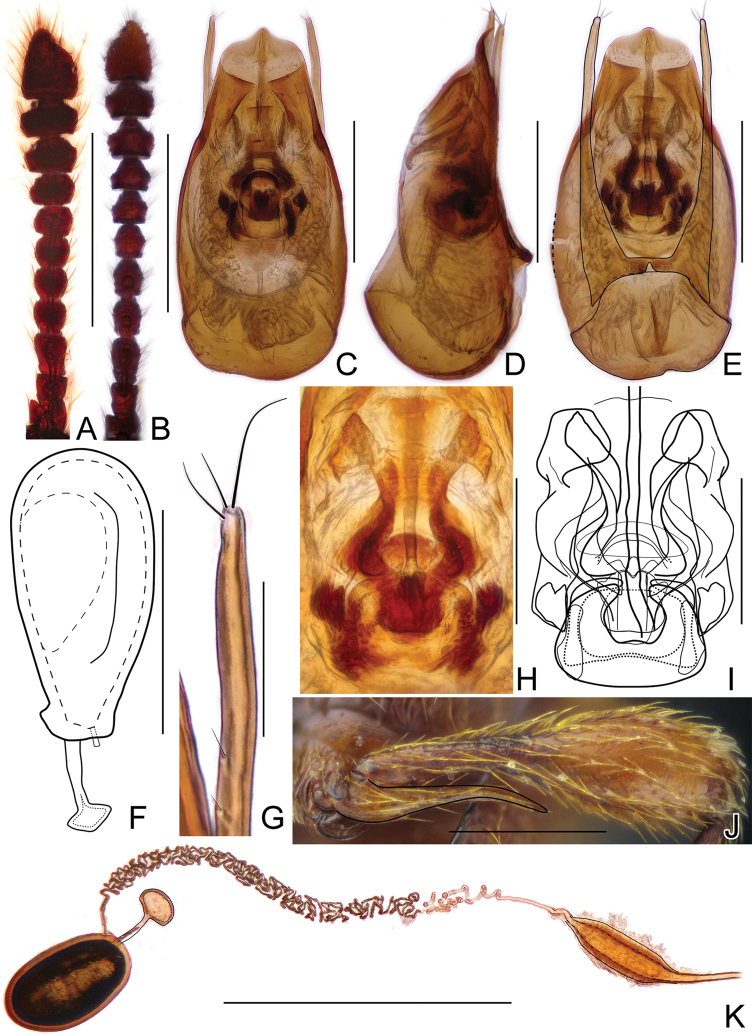
Diagnostic characters of *Horaeomorphus* species. (**A, F** *Horaeomorphus* sp. **B–E, G–K** *Horaeomorphus chinensis*) **A** Left antenna of male, in dorsal view **B** Left antenna of female, in dorsal view **C** Aedeagus, in ventral view **D** Same, in lateral view **E** Same, in dorsal view **F** Spermatheca, in lateral view **G** Apical portion of paramere, enlarged **H** Endophallus, enlarged, in ventral view **I** Same, schematic **J** Left metatrochanter of male, in ventral view **K** Spermatheca with bursa copulatrix, in lateral view. Scale bars: 0.5 mm (**A, B**); 0.2 mm (**C, D, E**); 0.1 mm (**F, H, I**); 0.04 mm (**G**); 0.3 mm (**J, K**).

**Figure 7.**
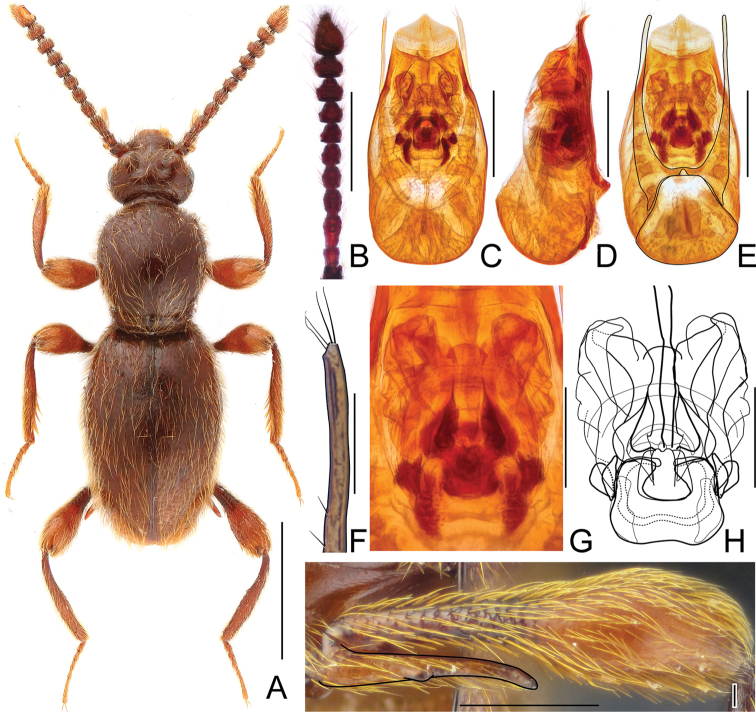
*Horaeomorphus pengzhongi* sp. n. **A** male, dorsal habitus **B** Right antenna of male, in dorsal view **C** Aedeagus, in ventral view **D** Same, in lateral view **E** Same, in dorsal view **F** Apical portion of paramere, enlarged **G** Endophallus, enlarged, in ventral view **H** Same, schematic **I** Left metatrochanter of male, in ventral view. Scale bars: 1 mm (**A**); 0.5 mm (**B**); 0.2 mm (**C, D, E**); 0.04 mm (**F**); 0.1 mm (**G, H**); 0.3 mm (**I**).

Female. Unknown.

#### Comments.

*Horaeomorphus pengzhongi* is similar to many congeners (see comments of *Horaeomorphus chinensis*); the relatively larger body (3.00 mm), elongated metatrochanters each with the ventral expansion in male, each paramere with two subapical setae and structures of endophallus are clearly different and can be used to identify this new species.

#### Distribution.

Southern China: Yunnan.

#### Etymology.

This species is dedicated to Zhong Peng, one of the collectors of the type specimen.

### Horaeomorphus biwenxuani

Taxon classificationAnimaliaColeopteraStaphylinidae

D.-Y. Zhou & S.-J. Zhang sp. n.

http://zoobank.org/E5D8A881-C40E-4321-BAAE-415074D29AEF

Fig. 8

#### Type material

(1 ♂)**. Holotype: CHINA**: ♂, labeled ‘China: Xizang Prov., Cuona County [错那县], Lexiang [勒乡], alt. 2500m, 15.vii.2012, Wen-Xuan Bi leg.’.

#### Diagnosis.

*Horaeomorphus biwenxuani* can be readily separated from all other congeners by its moderately large (2.78 mm) and elongate body, small pronotum lacking basal groove, with a row of three dorsal pits, subtriangular metatrochanter with distal edge produced into a short acute spine, and slender aedeagus with a complicated and strongly asymmetrical endophallus.

#### Description.

**Figure 8.**
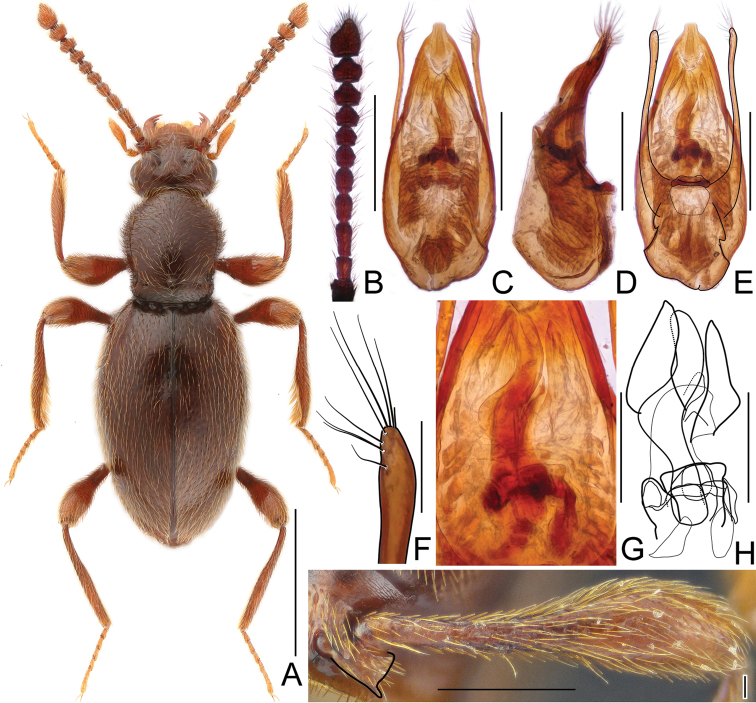
*Horaeomorphus biwenxuani* sp. n. **A** male, dorsal habitus **B** Left antenna of male, in dorsal view **C** Aedeagus, in ventral view **D** Same, in lateral view **E** Same, in dorsal view **F** Apical portion of paramere, enlarged **G** Endophallus, enlarged, in ventral view **H** Same, schematic **I** Left metatrochanter of male, in ventral view. Scale bars: 1 mm (**A**); 0.5 mm (**B**); 0.2 mm (**C, D, E**); 0.04 mm (**F**); 0.1 mm (**G, H**); 0.3 mm (**I**).

Male. BL 2.78 mm; body (Fig. 8A) large, slightly convex, dark reddish-brown, legs and palpi slightly lighter. Head broadest at large, finely faceted and moderately convex eyes, HL 0.41 mm, HW 0.58 mm; tempora rounded, about as long as length of eye in dorsal view; vertex strongly transverse and weakly convex, with pair of small but distinct pits located near posterior margins of supra-antennal tubercles; frons weakly convex; supra-antennal tubercles strongly raised. Punctation on vertex and frons sparse, small but distinct; setae moderately long, sparse. Antennae (Fig. 8B) short, AnL 1.18 mm, relative lengths of antennomeres: 0.9 : 1.0 : 1.6 : 1.3 : 1.1 : 1.0 : 0.8 : 0.9 : 1.0 : 1.1 : 1.7. Pronotum short, longer than wide, widest slightly behind anterior fourth, PL 0.77 mm, PWm 0.65 mm, PWb 0.53 mm; pronotal margin rounded near anterior 2/3, then nearly straight up to sub-basal constriction; base with row of three dorsal pits and pair of lateral impressions located in constriction. Punctation on disc sparse and fine; dorsal surface glossy; setation moderately long. Elytra elongate, more convex than pronotum, distinctly impressed in middle at about anterior third; widest near anterior 2/5, narrowing toward apices. EL 1.59 mm, EW 0.99 mm, EI 0.59. Humeral calli distinct. Punctures fine, more distinct than those on pronotum, sharply marked and separated by spaces 3–4× as wide as puncture diameters; setation moderately dense. Hindwings fully developed. Metatrochanter (Fig. 8I) short, subtriangular, distal edge produced into short acute spine. Aedeagus (Fig. 8C–E) slender, AeL 0.55 mm; endophallus (Fig. 8G–H) strongly asymmetrical, with curved axial component protruding from posterior complicated structure surrounded by two lateral Λ-shaped structures; parameres (Fig. 8F) very slender with broadened apical parts, slightly shorter than median lobe, each with nine apical and subapical setae.

Female. Unknown.

#### Comments.

This new species with remarkably long legs has subtriangular metatrochanters in males, each with a sharp distal edge. This unique character can be found also in all four known Nepalese congeners: *Horaeomorphus obrus* Vít, 2004, *Horaeomorphus deharvengi* Vít, 2004, *Horaeomorphus himalayensis* Franz, 1974 and *Horaeomorphus nepalensis* Franz, 1973 (Franz 1974; Vít 2004), but so far has not been recorded in *Horaeomorphus* outside the Himalayas. However, a relatively small body (2.78 mm; among Himalayan species only *Horaeomorphus deharvengi* can be smaller than 3mm) and strongly asymmetrical endophallus are clearly different from characters of the Nepalese species. An asymmetrical endophallus also occurrs in *Horaeomorphus deformatus* Jałoszyński, 2006 (W Malaysia: Kuala Terengganu), *Horaeomorphus pseudosabahensis* Jałoszyński, 2006 (E Malaysia: Sabah, Sarawak) and *Horaeomorphus minor* Jałoszyński, 2009 (the Philippines: Bukidnon, Mindanao), but its structure is distinctly different from that in *Horaeomorphus biwenxuani*.

#### Distribution.

Western China: Xizang.

#### Etymology.

This species is dedicated to Wen-Xuan Bi, who collected the type specimen.

### Horaeomorphus hujiayaoi

Taxon classificationAnimaliaColeopteraStaphylinidae

D.-Y. Zhou & S.-J. Zhang sp. n.

http://zoobank.org/85FD7AC1-06A4-41EA-8F3D-B8A2E2FB221C

Fig. 9

#### Type material

(1 ♂)**. Holotype: CHINA**: ♂, labeled ‘China: Guangxi Prov., Jinxiu County [金秀县], Mt.Lianhuashan [莲花山], alt. 1000–1150m, 30.vii.2011, Jia-Yao Hu leg.’.

#### Diagnosis.

*Horaeomorphus hujiayaoi* can be readily separated from all other congeners by its moderately large (2.53 mm), short pronotum with five basal pits connected by a shallow groove, unmodified metatrochanters, median lobe of aedeagus with a blade-shaped, asymmetrical apex bent at an obtuse angle in relation to the long axis of aedeagus and asymmetrical parameres each with ten apical and subapical setae.

#### Description.

**Figure 9.**
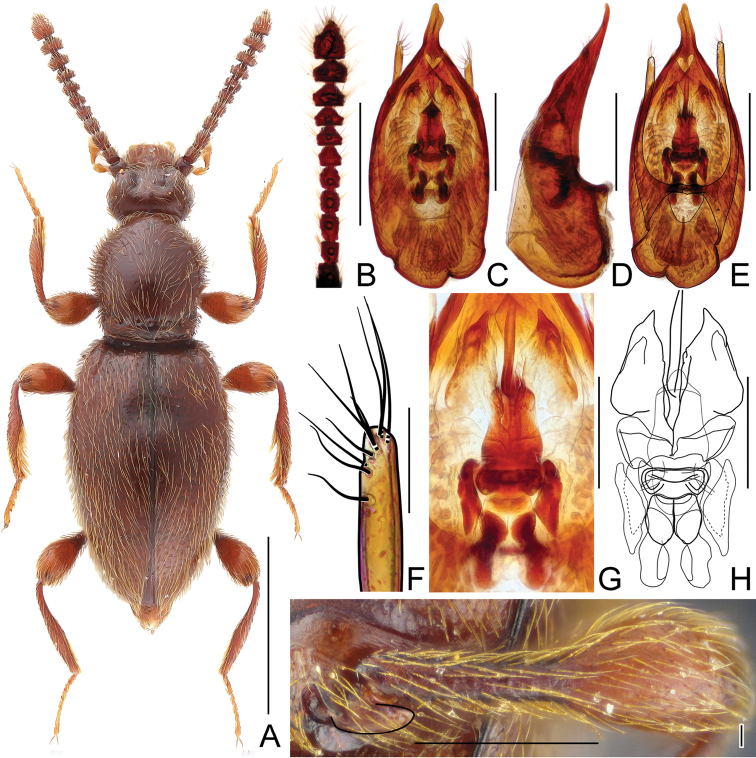
*Horaeomorphus hujiayaoi* sp. n. **A** male, dorsal habitus **B** Left antenna of male, in dorsal view **C** Aedeagus, in ventral view **D** Same, in lateral view **E** Same, in dorsal view **F** Apical portion of paramere, enlarged **G** Endophallus, enlarged, in ventral view **H** Same, schematic **I** Left metatrochanter of male, in ventral view. Scale bars: 1 mm (**A**); 0.5 mm (**B**); 0.2 mm (**C, D, E**); 0.04 mm (**F**); 0.1 mm (**G, H**); 0.3 mm (**I**).

Male. BL 2.53 mm; body (Fig. 9A) large, flattened, dark reddish-brown, legs and palpi slightly lighter. Head broadest at large, finely faceted, and moderately convex eyes, HL 0.36 mm, HW 0.50 mm; tempora rounded but not bent, about as long as length of eye in dorsal view; vertex strongly transverse and weakly convex, with pair of small but distinct pits located near posterior margins of supra-antennal tubercles; frons weakly convex; supra-antennal tubercles strongly raised. Punctation on vertex and frons sparse, small but distinct; setae moderately long, sparse. Antennae (Fig. 9B) short, AnL 1.01 mm, relative lengths of antennomeres: 1.2 : 1.0 : 1.3 : 1.0 : 1.0 : 1.0 : 0.9 : 0.9 : 1.0 : 1.0 : 1.9. Pronotum inversely subtrapezoidal , flattened, longer than wide, widest near middle, PL 0.76 mm, PWm 0.61 mm, PWb 0.53 mm; anterior margin rounded; lateral margins rounded near anterior 2/3, then nearly straight up to sub-basal constriction; base with five pits connected by shallow groove. Punctation on disc sparse and fine; dorsal surface glossy; setation moderately long. Elytra elongate, more convex than pronotum, distinctly impressed in middle at about anterior third; widest slightly before middle, narrowing toward apices. EL 1.41 mm, EW 0.93 mm, EI 1.51. Humeral calli distinct. Punctures fine, more distinct than those on pronotum, especially on impressed area, sharply marked and separated by spaces 2–4× as wide as puncture diameters; setation moderately dense. Hindwings fully developed. Metatrochanter (Fig. 9I) unmodified. Mesotibiae slightly curved and with inner margin expanded near middle to form broad subtriangular tooth. Aedeagus (Fig. 9C–E) moderately elongate, median lobe with a strongly asymmetrical blade-shaped apex bent at obtuse angle in relation to long axis of aedeagus, AeL 0.59 mm; endophallus (Fig. 9G–H) with complicated system of variously sclerotized structures; parameres (Fig. 9F) asymmetrical, of unequal lengths, each with ten apical and subapical setae.

**Figure 10.**
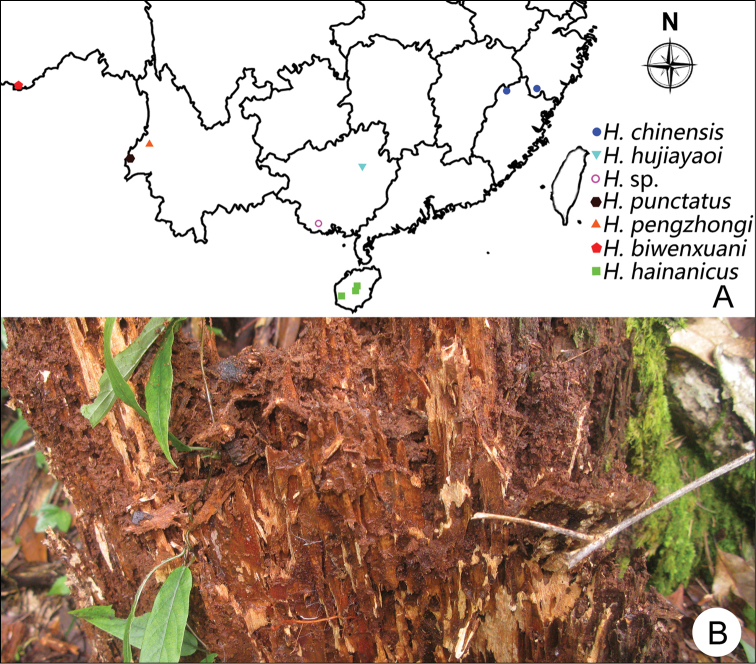
**A** Distribution of *Horaeomorphus* in mainland China **B** Habitat of *Horaeomorphus chinensis* at Guadun.

Female. Unknown.

#### Comments.

This new species has the aedeagal median lobe with an apical blade-shaped projection bent to the left in ventral view and parameres of unequal lengths, characters shared with *Horaeomorphus obrus* Vít, 2004 (Nepal: Janakpur, Bagmati). *Horaeomorphus hujiayaoi* can be unambiguously separated from its Nepalese congener by the much smaller body (2.53 mm vs 4.3 mm in *Horaeomorphus obrus*) and different structures of the endophallus.

#### Distribution.

Southern China: Guangxi.

#### Etymology.

This species is dedicated to Jia-Yao Hu, who collected the type specimen.

### Key to species of *Horaeomorphus* in mainland China

**Table:** 

1	Basal pits on pronotum connected by a shallow and barely notable groove	**2**
–	Basal pits on pronotum connected by a deep and distinct groove	**3**
2	Pronotum broad, nearly circular; elytral punctures coarse, sharply marked and separated by spaces as wide as puncture diameters	***Horaeomorphus punctatus* sp. n.** (Yunnan: Yingjiang)
–	Pronotum small; elytral punctation fine and sparse, elytra glossy	***Horaeomorphus biwenxuani* sp. n.** (Xizang: Cuona)
3	Pronotum with five basal pits	**4**
–	Pronotum with three basal pits	**5**
4	Body slender and flattened, EI > 1.5	***Horaeomorphus hujiayaoi* sp. n.** (Guangxi: Jinxiu)
–	Body stout and convex, EI < 1.4	***Horaeomorphus* sp.** (Guangxi: Shangsi)
5	BL < 2.5 mm; punctation of pronotal disc and elytra dense and coarse	***Horaeomorphus chinensis* Franz, 1985** (N.Fujian, S.Fujian)
–	BL > 2.5 mm; punctation of pronotal disc and elytra sparse and fine, surface glossy	**6**
6	Pronotum inversely subtrapezoidal, its lateral margins sharply bent at anterior third and strongly constricted near posterior fourth	***Horaeomorphus hainanicus* sp.n.** (Hainan)
–	Pronotum oval, its anterior margin and lateral margins evenly rounded together	***Horaeomorphus pengzhongi* sp.n.** (Yunnan: Tengchong)

## Supplementary Material

XML Treatment for Horaeomorphus chinensis

XML Treatment for Horaeomorphus sp.

XML Treatment for Horaeomorphus hainanicus

XML Treatment for Horaeomorphus punctatus

XML Treatment for Horaeomorphus pengzhongi

XML Treatment for Horaeomorphus biwenxuani

XML Treatment for Horaeomorphus hujiayaoi
